# Phytest: quality control for phylogenetic analyses

**DOI:** 10.1093/bioinformatics/btac664

**Published:** 2022-10-07

**Authors:** Wytamma Wirth, Simon Mutch, Robert Turnbull, Sebastian Duchene

**Affiliations:** Peter Doherty Institute for Infection and Immunity, University of Melbourne, Melbourne 3010, Australia; Melbourne Data Analytics Platform, University of Melbourne, Melbourne 3010, Australia; Melbourne Data Analytics Platform, University of Melbourne, Melbourne 3010, Australia; Peter Doherty Institute for Infection and Immunity, University of Melbourne, Melbourne 3010, Australia

## Abstract

**Motivation:**

The ability to automatically conduct quality control checks on phylogenetic analyses is becoming more important with the increase in genetic sequencing and the use of real-time pipelines e.g. in the SARS-CoV-2 era. Implementations of real-time phylogenetic analyses require automated testing to make sure that problems in the data are caught automatically within analysis pipelines and in a timely manner. Here, we present Phytest (version 1.1) a tool for automating quality control checks on sequences, trees and metadata during phylogenetic analyses.

**Results:**

Phytest is a phylogenetic analysis testing program that easily integrates into existing phylogenetic pipelines. We demonstrate the utility of Phytest with real-world examples.

**Availability and implementation:**

Phytest source code is available on GitHub (https://github.com/phytest-devs/phytest) and can be installed via PyPI with the command ‘pip install phytest’. Extensive documentation can be found at https://phytest-devs.github.io/phytest/.

**Supplementary information:**

Supplementary data are available at *Bioinformatics* online.

## 1 Introduction

Phylogenetics is increasingly performed in automated pipelines, run with increasing frequency as sequence data becomes more readily available [e.g. during the SARS-CoV-2/coronavirus disease 2019 (COVID-19) pandemic]. The frequency of runs and complexity of these pipelines can result in the introduction of erroneous data causing failures, or worse, incorrect results and conclusions. Manually checking analyses for errors can be repetitive and laborious, wasting researchers time. While automated testing software exists for code, no such testing frameworks are available to easily test for errors in phylogenetic analyses.

Here, we present Phytest (version 1.1.0) a tool for automating quality control checks on sequence, phylogenetic trees and associated metadata files during phylogenetic analyses. Phytest ensures that phylogenetic analyses meet user-defined quality control standards. Phytest can be installed from the Python Package Index (PyPI) using the command pip install phytest.

## 2 Implementation and usage

**Fig. 1.**
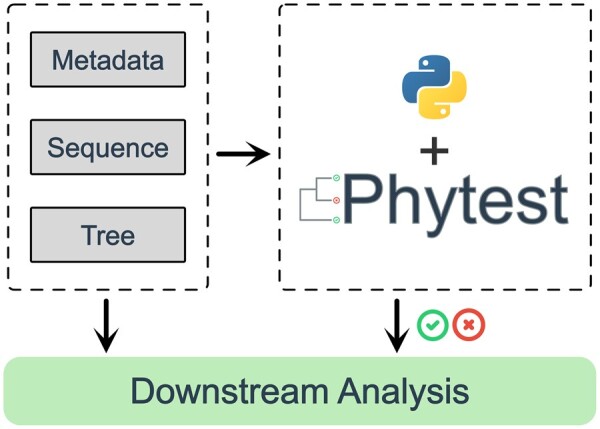
Conceptual figure of Phytest workflow. Sequence, tree and metadata files from a phylogenetic analyses are used as inputs to Phytest. Tests written in Python are run against these data structures using Phytest to determine if downstream analysis should proceed

Phytest is based on the popular Python testing framework Pytest (Krekel *et al.*, 2004). It allows users to write tests for their phylogenetic analyses the same way they write tests for their code (Fig. 1). Phytest has been developed as a command-line interface (CLI), Python module and Pytest plugin, providing multiple methods of invocation. It provides many convenient helper functions for testing phylogenetic analyses including methods for testing sequences, alignments, trees and metadata files.

Phytest is easily extendable and provides a simple interface for writing custom phylogenetic tests. The interface follows the Pytest model of testing i.e. tests are defined as Python functions containing assert statements that are collected and evaluated at run-time. Tests that fail are captured and reported to the user allowing for repeatable and automated testing.

Phytest injects special fixtures into test functions, allowing for easy evaluation and testing of phylogenetic data structures. These fixtures provide the standard Biopython (sequences, alignments and trees) and Pandas (metadata) class methods as well as special assert methods for testing these data structures (Cock *et al.*, 2009; McKinney *et al.*, 2010). For example, the Phytest Sequence class implements the method Sequence.assert_percent_GC. Calling this method on the fixture object with the expected GC-content e.g. sequence.assert_percent_GC(38) will raise an error if the percent of G and C nucleotides in the sequence is not equal to 38%. Many methods also provide maximum and minimum arguments so the upper and lower bounds can be tested e.g. sequence.assert_percent_GC(min = 30, max = 40).

All Phytest assert methods also provide a warning flag, e.g. sequence.assert_percent_GC(38, warn=True) causing the method to raise a warning instead of an error if the test fails. In an automated pipeline, this provides a way to inform the user of potential problems without causing the pipeline to fail. The warning flag can be set automatically by calling the method with the warn_ prefix instead of assert_ e.g. sequence.warn_percent_GC(38). See the documentation for a full list of built-in assert methods (https://phytest-devs.github.io/phytest/reference.html).

Phylogenetic tree, sequence and metadata files are passed from the command line, allowing test re-usability on different files. The Phytest CLI requires a path to the file containing user-defined tests and has optional flags for specifying sequence/alignment, tree and metadata files e.g. phytest test.py --sequence sequences.fasta --tree tree.newick --data metadata.csv. Alternative file formats can be specified with --sequence-format, --tree-format and --data-format flags, supported formats include those supported by Biopython and comma/tab-separated values and Excel formats for metadata. Using the Phytest --report flag will generate a detailed HTML report of test results.

## 3 Examples

We provide several examples of how Phytest can integrate into standard phylogenetic analyses and scenarios as separate repositories in the phytest-devs GitHub organization (https://github.com/phytest-devs). These include Phytest for quality control in a Nextstrain/Snakemake pipeline (Hadfield *et al.*, 2018; Mölder *et al.*, 2021) (https://github.com/phytest-devs/phytest-nextstrain-example), Testing phylogenetic data with Continuous Integration features on GitHub (https://github.com/phytest-devs/phytest-continuous-testing-example) and using Phytest to test for temporal signal (https://github.com/phytest-devs/phytest-temporal-signal-example).

### 3.1 Temporal signal example

Temporal signal is an important prerequisite for estimating evolutionary rates and timescales (Rieux and Balloux, 2016). A data set with temporal signal is one in which the sampling time span captures sufficient genetic variation to allow for estimates of the evolutionary rate (also known as the molecular clock rate). Analyses of temporal signal can also be useful to detect problematic sequences, such as those with sequencing errors or mislabeled dates, before fitting a molecular clock and estimating evolutionary rates and dates, such as when using BEAST (Bouckaert *et al.*, 2019; Drummond and Rambaut, 2007). A repository containing the code for this example can be found at https://github.com/phytest-devs/phytest-temporal-signal-example. Here, we use data from the TempEst tutorial https://beast.community/tempest_tutorial. TempEst is a useful program for performing temporal signal analysis, however, it is not possible to easily automate the TempEst graphical user interface (Rambaut *et al.*, 2016). Internally, Phytest uses TimeTree to perform a root-to-tip regression, allowing users to automate temporal signal testing (Sagulenko *et al.*, 2018). The Tree.assert_root_to_tip method is used for testing temporal signal and provides arguments for testing the coefficient of determination (*R*^2^), regression slope (a crude estimate of the evolutionary rates) and root date.

## 4 Conclusions

Phytest is a flexible and powerful tool for reproducibility and automation. New data are often incorporated or manually edited when developing or optimizing a phylogenetic analysis pipeline. Phytest ensures that analyses meet user-specified standards each time they are run. We believe Phytest will be increasingly useful as the use of automated phylogenetic pipelines increases, especially in the field of real-time phylogenetic analysis.

## Funding

This work was supported by the Australian Research Council [DE190100805] and Australian National Health and Medical Research Council (NHMRC) [APP1157586].

*Conflict of Interest*: none declared.
